# Carbamazepine modulates the spatiotemporal activity in the dentate gyrus of rats and pharmacoresistant humans in vitro

**DOI:** 10.1002/brb3.463

**Published:** 2016-04-15

**Authors:** Natalie L. M. Cappaert, Taco R. Werkman, Nuria Benito, Menno P. Witter, Johannes C. Baayen, Wytse J. Wadman

**Affiliations:** ^1^ Swammerdam Institute for Life Sciences – Center for NeuroScienceUniversity of AmsterdamAmsterdamThe Netherlands; ^2^ Department of Anatomy and NeuroscienceInstitute for Clinical and Experimental NeurosciencesVU University Medical CenterAmsterdamThe Netherlands; ^3^ Department of NeurosurgeryVU University Medical CenterAmsterdamThe Netherlands

**Keywords:** Antiepileptic drug, brain slices, hippocampus, temporal lobe epilepsy, voltage‐sensitive dye imaging

## Abstract

**Introduction:**

Human hippocampal tissue resected from pharmacoresistant epilepsy patients was investigated to study the effect of the antiepileptic drug CBZ (carbamazepine) and was compared to similar experiments in the hippocampus of control rats.

**Methods:**

The molecular layer of the DG (dentate gyrus) of human epileptic tissue and rat nonepileptic tissue was electrically stimulated and the evoked responses were recorded with voltage‐sensitive dye imaging to characterize the spatiotemporal properties.

**Results:**

Bath applied CBZ (100 *μ*mol/L) reduced the amplitude of the evoked responses in the human DG, albeit that no clear use‐dependent effects were found at frequencies of 8 or 16 Hz. In nonepileptic control DG from rats, CBZ also reduced the amplitude of the evoked response in the molecular layer of the DG as well as the spatial extent of the response.

**Conclusions:**

This study demonstrates that CBZ still reduced the activity in the DG, although the patients were clinically diagnosed as pharmacoresistant for CBZ. This suggests that in the human epileptic brain, the targets of CBZ, the voltage‐gated Na^+^ channels, are still sensitive to CBZ, although we used a relative high concentration and it is not possibility to assess the actual CBZ concentration that reached the target in the patient. We also concluded that the effect of CBZ was found in the activated region of the DG, quite comparable to the observations in the nonepileptic rat.

## Introduction

MTLE (Mesial temporal lobe epilepsy) is the most common form of epilepsy in adults (Engel et al. 1989). In most patients with MTLE, seizures can be controlled with AEDs (antiepileptic drugs), but 30% of the patients are nonresponsive or show unbearable side‐effects (Kwan et al. 2010). A successful alternative treatment for these pharmacoresistant patients is epilepsy surgery, where the epileptic focus and some of the surrounding brain tissue are surgically removed (Wiebe et al. 2001; Engel et al. 2012). It is mostly unknown why AEDs are not effective in these patients. In this study, acute brain slices of the excised tissue, that became available after epilepsy surgery, were used to study the responsiveness to CBZ (carbamazepine), one of the most commonly used AEDs (Musshoff et al. 2000; Straub et al. 2000, 2001; Gorji et al. 2002).

Carbamazepine predominantly interacts with voltage‐dependent Na^+^ channels by binding to the inactivated state (Rogawski and Loscher 2004; Qiao et al. 2014). In this way, CBZ inhibits Na^+^ channels in a use‐dependent manner. CBZ only moderately blocks Na^+^ channels at hyperpolarized membrane potential. More pronounced effects are achieved at depolarized membrane potentials and its largest effects are exerted during high‐frequency activity (Ragsdale and Avoli 1998; Vreugdenhil and Wadman 1999; Sun et al. 2006).

There are several reports on CBZ modulation of Na^+^ channels in pharmacoresistant patients, but the results are inconclusive. CBZ reduced the amplitude of the Na^+^ currents in isolated human DG (dentate gyrus) granule cells by inducing a shift in the voltage dependence of steady‐state inactivation (Reckziegel et al. 1999). However, CBZ failed to block the use‐dependent Na^+^ channels in DG granule cells of MTLE patients (Remy et al. 2003). Moreover, 100 *μ*mol/L CBZ did reduce epileptiform activity (induced by hilar stimulation in the presence of an elevated extracellular potassium concentration) in the DG granule cell layer of human epileptic hippocampal slices measured with extracellular recordings (Jandova et al. 2006).

Moreover, extracellular recordings in the DG granule cell layer of human epileptic hippocampal slices showed a reduced epileptiform activity in the presence of 100 *μ*mol/L CBZ in response to hilar stimulation in the presence of an elevated extracellular potassium concentration (Jandova et al. 2006).

Most in vitro studies investigated the effect of CBZ at a single location or neuron in the slice. However, CBZ may influence the spatial recruitment of brain tissue in response to electrical stimulation. The spatiotemporal dynamics of epileptic activity in human tissue was mainly investigated in the neocortex (Albowitz et al. 1998; Kohling et al. 1999; Straub et al. 2003) and to some degree in the hippocampus (Cohen et al. 2002). Electrical stimulation of human neocortical slices recruited a larger area of evoked activity when bathed in epileptogenic medium than in control medium (Albowitz et al. 1998; Kohling et al. 1999).

In this study, we use VSDi (voltage‐sensitive dye imaging) to investigate the spatial recruitment of electrically evoked responses in excised hippocampal brain tissue of pharmacoresistant epilepsy patients and in hippocampal DG slices of control rats. Moreover, the effect of CBZ on the spatial dynamics of electrically evoked responses was studied in both preparations.

## Experimental Procedure

### Epilepsy patients and control rats

Hippocampal tissue from three patients suffering from pharmacoresistant MTLE was obtained. Detailed information about the patients' seizure history, AEDs, and histopathological findings are given in Table 1. All patients had been treated with several AEDs before surgery and all patients received or were receiving CBZ before the surgery. The study of tissue was approved by the local ethics committee and informed consent was obtained from all patients.

**Table 1:** Data of patients

Case	1	2	3
Gender	Female	Female	Male
Age at onset (years)	14	17	6
Age at surgery (years)	36	20	18
AED	**CBZ**, MZP, OCBZ, PHB, VGB, VPA	**CBZ**, GBP, LEV, LTG, TPM, VPA,	**CBZ**, GBP, LTG, VPA
Seizure type	SPS, CPS	SPS	CPS
Site of resection	Right	Left	Right
Seizure frequency (/y)	48	1000	216
MRI	Lesion	MTS	MTS
Lesion	Ganglioglioma/cystic astrocytoma/DNET		
Pathology	Tumor/lesion	MTS	MTS
Specified pathology	DNET (WHO I)	HS (W 3/4)	HS (W 3/4)

Antiepileptic drugs (AED): CBZ, carbamazepine; GBP, gabapentine; LEV, levetiracetam; LTG, lamotrigine; MZP, mirtazapine; OCBZ, oxcarbazepine; PHB, phenobarbital; TPM, topiramate; VGB, vigabatrine; VPA, valproic acid;

Seizure types: CPS, complex partial seizure; SPS, simple partial seizure;

Pathology: DNET, dysembryoplastic neuroepithelial tumor; MTS, medial temporal lobe sclerosis; W, Wyler score.

Hippocampal–entorhinal cortex slices were prepared from 100 to 150 g male Wistar rats (Harlan, Zeist, the Netherlands). Care and use of the animals were approved by the Animal Care and Use Committee of the University of Amsterdam, and are in accordance with European guidelines.

### Slice preparation and VSD staining

After resection of the hippocampus in the operating room, a small part of the human hippocampus (approximately 1 cm^3^) was immediately stored in ice‐cold ACSF (artificial cerebral spinal fluid, containing (in mmol/L): 120 NaCl, 3.5 KCl, 1.3 MgSO_4_, 1.25 NaH_2_PO_4_, 2.5 CaCl_2_, 10 d‐glucose, and 25 NaHCO_3_) saturated with 95% O_2_ and 5% CO_2_ to set the pH at 7.4. After transport from the hospital to the laboratory (~45 min) in ice‐cold ACSF, slices of 400‐*μ*m thickness were obtained with a vibratome (Leica VT 1000S, Wetzlar, Germany). The slices were transferred to a holding chamber containing ACSF at room temperature and stained for 1 h with 0.003 mg/mL of the oxonol VSD, NK3630 (Hayashibara Biochemical Laboratories, Inc., Kankoh‐Shikiso, Okoyama, Japan). After the staining period, the slices were kept at room temperature in ACSF until they were placed in the recording chamber.

Rats were decapitated and the brains were quickly removed. The whole brain was placed for approximately 1 min into ice‐cold ACSF before dissecting the block that contained the hippocampus and the entorhinal cortex and horizontal slices of 400‐*μ*m thickness were prepared with a vibratome. After slicing, the slices were kept in ACSF at room temperature. Following an acclimatization period of 30 min, the slices were stained with 0.003 mg/mL NK3630 dye. After a 1‐h staining period, the slices were transferred to a holding chamber onto a LCR membrane filter (MilliPore membrane filter, FHLC02500, PTFE hydrophilic membrane with 0.45 *μ*m pore size, Millipore, Billerica, NA) placed on an ACSF‐filled well, where they were kept at room temperature in ACSF under a moistened mixture of 95% O_2_ and 5% CO_2_.

### Optical recordings

**Figure 1:**
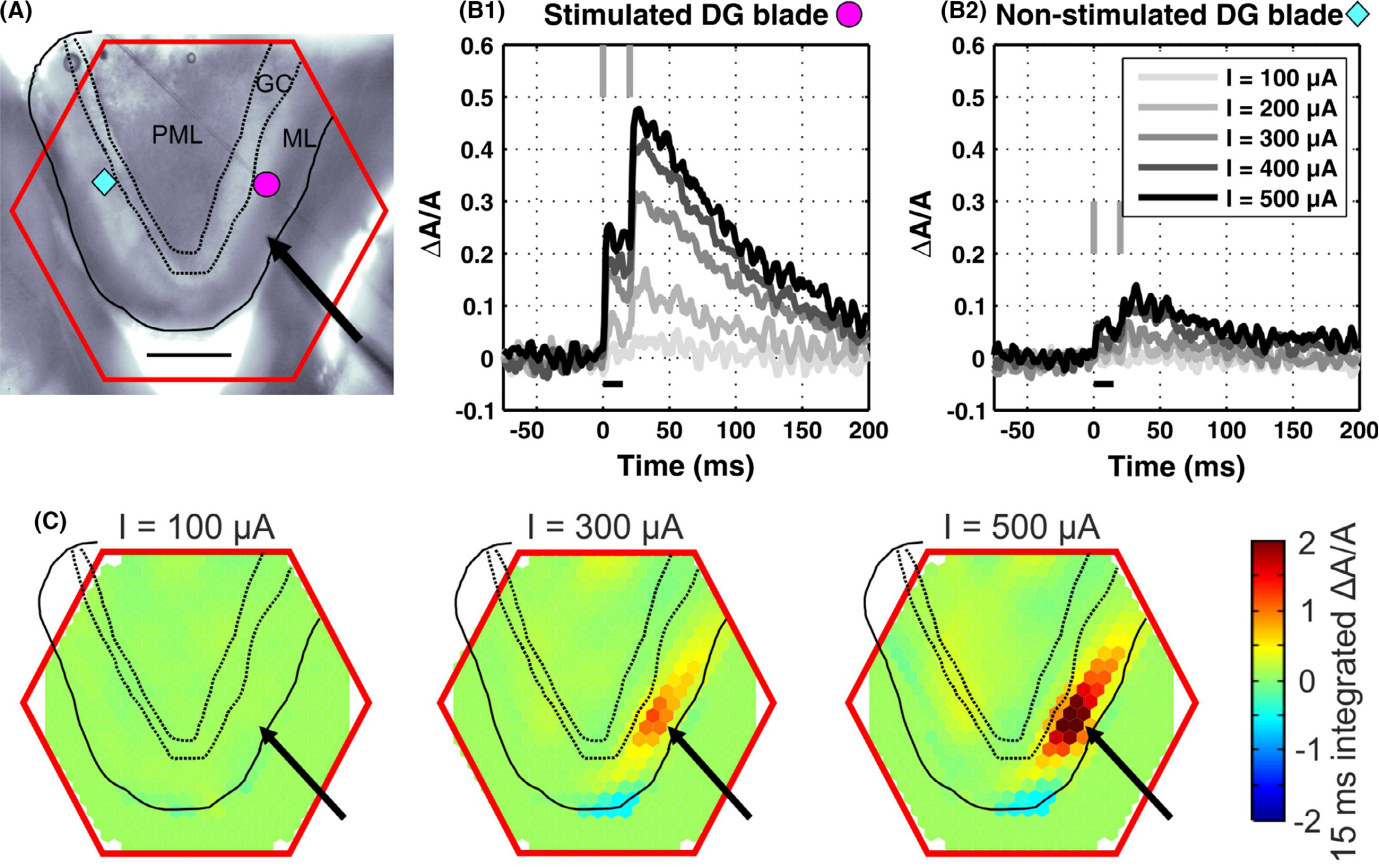
Input–output response of human dentate gyrus. (A) Image of the DG (dentate gyrus) area of a recorded human hippocampal slice (slice 1 of case 1, Table 1). The black solid line indicates the outline of the ML (molecular layer) and the dotted lines mark the boundaries of the GC (granule cell) layer. The polymorphic layer or hilus is indicated with PML. The red hexagonal overlay represents the outline of the photodiode array. The magenta circle and blue diamond indicate the VSDi recording sites shown in (B). The position of the bipolar stimulation electrode is indicated by the black arrow. The scale bar represents 1 mm. (B) Optical responses recorded in the DG of the human hippocampal slice shown in A to a double pulse stimulus (timing indicated by gray vertical lines) of 100–500 *μ*A, with an interpulse interval (IPI) of 20 msec. The traces are recorded in the ML layer of the DG and are an average of three photodiodes located in the stimulated blade (B1 indicated with a magenta circle in A) and in the nonstimulated blade of DG (B2 indicated with a blue diamond in A). (C) The responses presented in B are integrated from 0 to 15 msec (indicated by the black horizontal lines in B) after the first stimulus pulse for every VSDi recording site. These integrated values are represented in color code at the corresponding location of these recording sites in the photodiode array and are depicted for 100, 300, and 500 *μ*A stimulation. The black and red shapes are the outlines of the anatomical regions and photodiode array, respectively, as in A. The position of the bipolar stimulation electrode is indicated by black arrows.

In the recording chamber, the electrically evoked optical responses were recorded with a 464‐channel, hexagonally arranged, photodiode array (H‐469II Photodiode Array, WuTech Instruments, Gaithersburg, MD). A 5x objective (0.25 NA Fluar, Zeiss, Oberkochen, Germany) was used to project the image of the slice onto the array. A second optical channel in the microscope (Axioskop 2FS, Zeiss, Germany) allowed taking images of the slice (SPOT, Diagnostic Instruments, Stirling Heights, NJ) for offline superposition of the morphology on the diode recording sites. In Figure 1A, the hexagonal‐shaped area of the diode array is indicated as overlay on a human hippocampal slice. The slice was illuminated with 705 ± 5 nm (Jin et al., 2002). The signal from each diode was high‐pass filtered (>0.2 Hz), amplified, and then digitized at 1 kHz with a 12‐bit data acquisition board (DAP 3200a/415 Microstar Laboratories, Bellevue, WA). The data acquisition was controlled by a custom‐made program (for details, see Wu et al., 1999).

Each recording session started with a low‐gain recording of the absolute light level (A) in each diode, which was calculated from the a transient response obtained after opening the mechanical light shutter (Uniblitz, Rochester, NY). Subsequent recordings were made, when the light was continuously on, at the high gain setting. For each diode, the change in absorbance ΔA(*t*) was then normalized to absolute light level (A), under the assumption that that A did not vary over the time period of the actual recording. Each photo diode received light from many stained membranes in the field of view and is thus related to changes in membrane potential of several neurons. The resulting signal (ΔA(*t*)/A) reflects a population average, is called “activity” in this study. The voltage‐sensitive dye NK3630 indicates membrane depolarization as a decrease in relative absorption, which we represent as a positive signal.

### Field potential recording

Glass microelectrodes filled with ACSF were used to record extracellular field potentials in the granule cell layer of the DG. The field potentials were preamplified and sampled at 50 kHz. Field potential recordings were recorded with a computer‐controlled data acquisition system (NI‐USB‐ 6259, National Instruments, Austin, TX). The intertrial interval was 8 sec to allow return to stable baseline conditions. Field potentials were averaged over three trials.

Field potentials were analyzed using custom‐made software MATLAB (The MathWorks, Inc., Natick, MA). Recordings in the granule cell layer of DG displayed a typical extracellular positive‐going postsynaptic potential (fEPSP) reflecting the depolarization of the postsynaptic membrane. The amplitude of the fEPSP is defined as the most positive point in the fEPSP.

### Electrical stimulation

Electrical stimulations were applied with a bipolar stimulation electrode (60 *μ*m diameter isolated stainless steel wire, tips 50–100 *μ*m apart). Stimulation electrodes were placed under direct visual guidance. In the human slices, the stimulation electrode was placed in the outer portion of the molecular layer of the DG (Fig. 1A). In the rat slices, the electrode was located at the border of the molecular layer of the DG and the EC at the point where the perforant path enters the DG, thus stimulating entorhinal input to the DG (Fig. 3A). Bipolar, biphasic stimulation pulses of 200 *μ*sec duration were applied through a constant‐current isolation unit. In the VSD experiments in human brain slices, the stimulation current was varied between 0 and 600 *μ*A. For each rat brain slice, the threshold stimulation intensity that evoked a response (I = 0%) and the stimulation intensity that saturated the response (I = 100%) were determined and used to standardize the stimulation over all tested slices. The average threshold stimulation intensity was 61 ± 21 *μ*A and the stimulation intensity that saturated the response was 771 ± 110 *μ*A (*n* = 7). The intertrial interval was at least 8 sec to allow return to stable baseline conditions. On some occasions, double pulses with a 20‐msec interval were used. Repetitive stimuli consisting of 10 identical stimuli at 8 or 16 Hz were applied.

### Data analysis

The optical responses were analyzed using custom‐made software created with MATLAB. Individual trials of all realizations were visually inspected and only trials with a stable baseline and without excessive noise were chosen for further analysis. All reported responses were means of at least three realizations. Instrumentation offset was removed for each individual diode signal by subtracting the mean value over a 15‐msec window preceding the stimulus.

Data traces displayed as a function of time (e.g., Fig. 1B) were averaged over 3–4 adjacent diode channels to best reflect the response from a specific region. The spatial dynamics of the evoked responses were visualized with false color maps (e.g., Fig. 1C). The data used in the spatial representation was filtered in space with a two‐dimensional Gaussian filter with a kernel size of approximately one interdiode distance.

**Figure 2:**
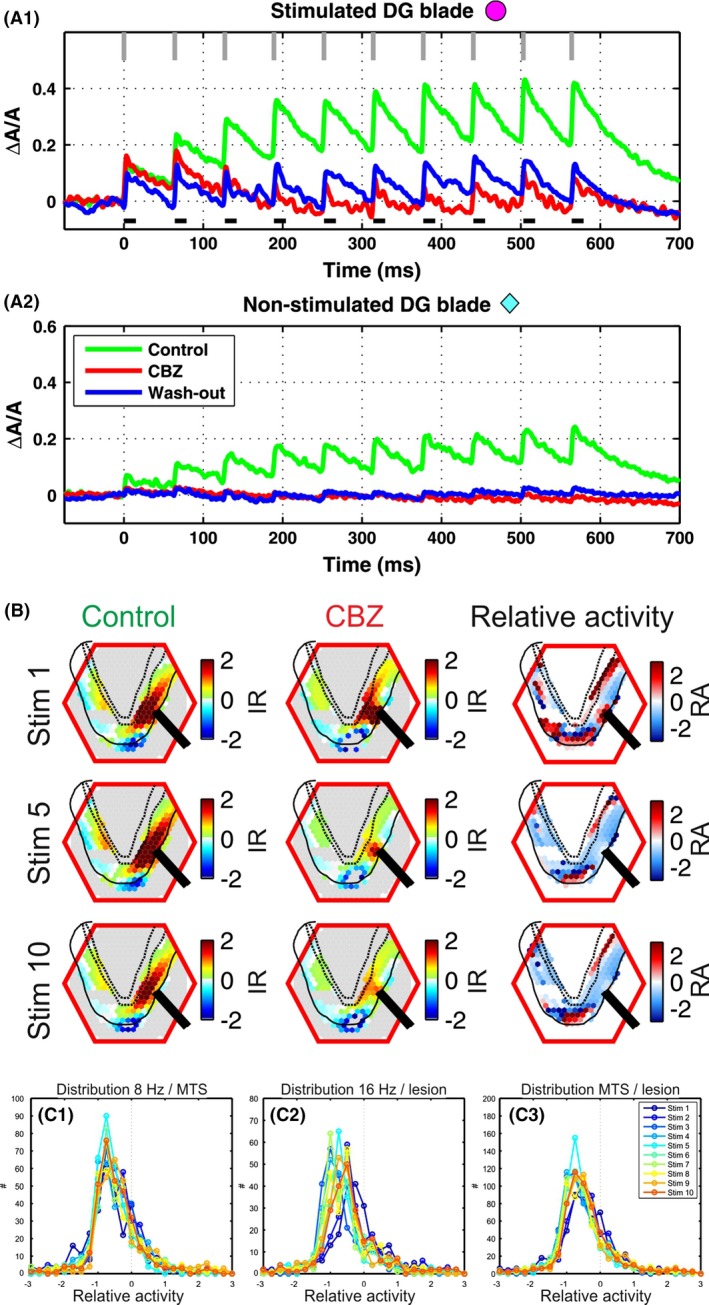
Effect of carbamazepine in human dentate gyrus. (A) The response of the DG (dentate gyrus) molecular layer evoked by 10 stimuli at 16 Hz of 500 *μ*A (vertical gray lines) recorded in the stimulated (A1) and nonstimulated blade (A2) of the DG (the same slice as is shown in Fig. 1), for the control situation (“Control”), after 20 min incubation with 100 *μ*mol/L CBZ (“CBZ”) and after 20 min wash‐out of CBZ (“Wash‐out”). (B) Spatial dynamics of the amplitude of the integrated response (IR) defined by the integral of the signal over the first 15 msec following each stimulus pulse (indicated by the black horizontal lines in A1) in the ROI (region of interest). The first two columns show the integrated response for the control and CBZ conditions described in A and for stimulus pulses (stim) 1, 5, and 10. The third column shows the normalized difference of the relative activity (RA) between the control and the CBZ condition (CBZ – Control/Control). (C) Distribution of the relative activity at 10 repeated stimuli, for six DG slices within a ROI, showing C1) the distribution for two slices of a patient with a lesion (16 Hz), C2) the distribution of four slices of two patients with MTS (8 Hz), and C3) the overall distribution of all slices (MTS + Lesion).

The parameter “integrated response” is the mean of the signal over a 15‐msec window, starting from the onset of the stimulation pulse. The resulting integrated response value was color coded as well and plotted at the designated location of the photodiode array to visualize the spatial dynamics of the response (e.g., Figs. 2B, 5B). During repetitive stimulation, the response to each stimulus is superimposed on the decay phase of the previous response (e.g., Fig. 2A). In this case, the integrated response was determined with respect to the signal value immediately before each stimulus.

Color‐coded plots were also used to compare the spatial dynamics of the relative activity between the control and the CBZ condition. The relative change in “integrated response” was calculated for each diode signal (e.g., Fig. 2B – third column). Blue colors correspond to a (local) reduction in activity by CBZ, while red colors indicate an increase.

Slices obtained from rat DG had a great similarity in shape, so that it was possible to spatially average the responses over slices. To that aim we defined seven fields within the DG of each hippocampal slice (Fig. 6A) and calculated the mean value of the “integrated response” for each field. These values were then averaged over slices (*n* = 7) and could also be presented in color code in the schematic DG, which contained the seven predefined fields (Fig. 6: B1 – 8 Hz and B2 – 16 Hz).

Student's *t*‐test and ANOVA with repeated measures were used to compare two or multiple groups. *P* < 0.05 was assumed to indicate a significant difference.

### Drugs

All pharmacological agents were prepared from stock solutions and dissolved in ACSF. CNQX disodium salt (Abcam Biochemicals, Cambridge, U.K.), and DL‐AP5 (Abcam Biochemicals) were dissolved in distilled water, with a resulting concentration of 200 *μ*mol/L CNQX and 40 *μ*mol/L AP5 in the ACSF. CBZ (Sigma‐Aldrich Chemie B.V., Zwijndrecht, the Netherlands) was dissolved in DMSO (dimethyl sulfoxide) solution, which resulted in a final concentration of 0.025% DMSO in the ACSF. The drug was washed‐in for 20 min. The time needed for the lipophilic CBZ to reach the maximal target concentration is not known, but Jandova and colleagues reported that the effective concentration is reached after 20 min of bath application (Jandova et al. 2006).

## Results

### Evoked responses in human epileptic dentate gyrus

In total, six slices of epileptic human tissues were gathered, two slices from one patient diagnosed with a lesion (case 1, Table 1) and four slices from two patients diagnosed with MTS (mesial temporal sclerosis; cases 2 and 3 Table 1). The outer portion of the DG molecular layer was electrically stimulated (Fig. 1A) and the evoked responses were recorded with VSD imaging (Fig. 1B and C). In a typical example for both lesion and MTS (slice 1 of case 1, see Table 1), the input/output relation with double pulses of increasing stimulus intensities (100–500 *μ*A, with steps of 100 *μ*A, interstimulus interval of 20 msec) is represented in the control condition with ACSF perfusion (Fig. 1B). The mean response in time for three adjacent photodiode channels is displayed for two locations in the molecular layer (Fig. 1A and B). The response amplitude in the nonstimulated blade of the DG was much smaller compared to the response in the stimulated blade. Typically, the amplitude of the evoked response increased with increasing stimulus intensity.

The spatial dynamics of the evoked response over the slice is shown in Figure 1C (slice 1 of case 1, Table 1). Stimulation with 100 *μ*A did not trigger a response, but increasing the stimulus intensity to 300 *μ*A evoked a response, mostly confined to the stimulated blade. Stimulating with increasing stimulus intensity increased the amplitude of the response near the stimulation electrode and further away. In all six slices, the stimulus‐evoked depolarization was strongest nearby the stimulation electrode and the depolarization gradually reduced at DG locations more distant from the stimulus electrode.

Next, we applied a stimulus train of 10 pulses at 8 or 16 Hz at 500 *μ*A. An example of the response in time is shown in Figure 2A (slice 1 of case 1, Table 1). The stimulus train at 16 Hz activated the DG in the molecular layer of both the stimulated blade and nonstimulated blade (Fig. 2A – “Control”). The amplitude of the response increased with increasing pulse number and in this particular case leveled out after the sixth pulse.

The integrated response over 15 msec was used to determine the spatial dimension of the evoked activity. The spatial dynamics in control ACSF is shown in Figure 2B (“Control”, slice 1 of case 1, Table 1) and the response evoked by the first, fifth, and 10th pulse produce a comparable activation pattern.

### Effect of carbamazepine in the human dentate gyrus

Bath application of 100 *μ*mol/L CBZ for 20 min did not affect the amplitude of the first response to the stimulus train in the stimulated blade close to the stimulation electrode in the example slice (Fig. 2A1, slice 1 of case 1), but the first response was reduced in the nonstimulated blade (Fig. 2A2). The response amplitude decreased in both blades following subsequent pulses in the stimulus train (Fig. 2A – “CBZ”). The spatial dimension of the first evoked response during CBZ application (Fig. 2B – “CBZ”) was comparable with the control situation (ASCF only – Fig. 2B “Control”), but the spatial dimension of the subsequent pulses was reduced. After wash‐out of CBZ for 30 min (Fig. 2A – “Wash‐out”), the amplitude of the responses did not fully return to the level of the control condition.

The relative activity between the control and the CBZ condition is calculated as the integrated value of the CBZ condition minus the integrated value of the control condition divided by the integrated value of the control condition (Fig. 2B – third column). The majority of the diode channels in the example had a smaller integrated activity in response to the first, fifth, and 10th pulse, indicating that CBZ reduced the evoked response.

**Figure 3:**
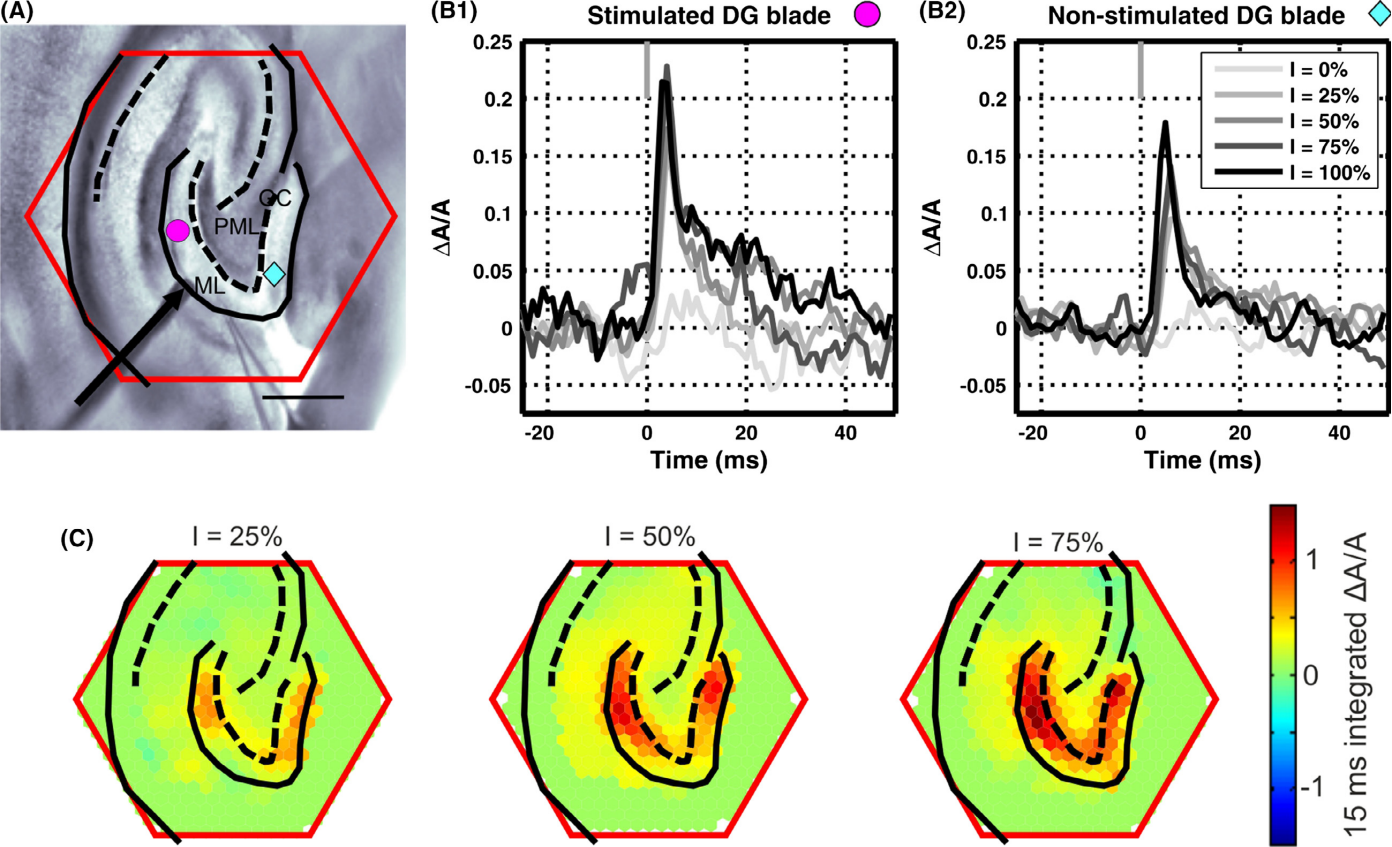
Input–output response of rat dentate gyrus. (A) Image of a recorded rat hippocampal slice. The black solid line indicates the outline of the molecular layer of the dentate gyrus and the outline of the Cornu ammonis. The dotted lines mark the granule cell layer and the pyramidal cell layer of CA3 and CA1. The polymorphic layer or hilus is indicated with PML. The red hexagonal overlay represents the outline of the photodiode array. The magenta circle and blue diamond indicate the VSDi recording sites represented in B. The position of the bipolar stimulation electrode is indicated by a black arrow. Scale bar indicates 1 mm. (B) Optical responses recorded in the DG of the rat hippocampal slice shown in A to a single pulse stimulus of 0–100% stimulus intensity. The traces are recorded in the ML layer of the DG and are an average of three channels which location is indicated with a magenta circle (B1; stimulated blade of DG) and a blue diamond (B2; nonstimulated blade of DG) in A. The gray vertical lines indicate the time of the electrical stimulation. (C) The responses presented in B are integrated from 0 to 15 msec after the start of the stimulus pulse for every VSDi recording site. These integrated values are represented in color code at the corresponding location of these recording sites in the photodiode array and are depicted for 25, 50, and 75% stimulation. The black and red shapes are the outlines of the anatomical regions and photodiode array, respectively, as in A.

To corroborate this CBZ effect for all six tested slices, we first defined the ROI (region of interest) by selecting the channels in the DG that responded to the stimulus in the control situation. Second, the integrated response to every stimulus pulse was calculated for all ROI channels of all slices in the control and CBZ condition. Finally, the relative activity for all the ROI channels was calculated and its distribution is plotted in Figure 2 (MTS/8 Hz – Fig. 2C1; Lesion/16 Hz – Fig. 2C2; all slices/lesion + MTS: Figs. 2C, 3C). All distributions are shifted to the left, which indicates that CBZ reduced the evoked response in the ROI of all tested slices. This CBZ effect on the integrated area was significant on all 10 pulses (Two‐way ANOVA with repeated measures performed on the data from the MTS + lesion, *P* < 0.01).

### Evoked responses in rat dentate gyrus

Voltage‐sensitive dye experiments in DG slices obtained from healthy rats (*n* = 7 slices from three rats, Fig. 3A) were performed to substantiate the results collected in the human slices. A typical example of the input–output relation was determined in the control situation with ACSF perfusion (Fig. 3B), showing the responses in time for the stimulated and nonstimulated blade in response to single pulses at intensities between 0 and 100% of the maximum stimulus intensity. Typically, the responses were detected in both blades following all stimulus intensities (Fig. 3C) and increasing the intensity of the stimulus enhanced the amplitude of the response in the molecular layer of the DG.

Application of 200 *μ*mol/L CNQX and 40 *μ*mol/L AP5 completely blocked the evoked responses, except for the responses recorded in the direct surrounding, the stimulation electrode (data not shown), indicating that the responses in the largest part of the molecular layer were a result of synaptically induced activation (Jackson and Scharfman 1996; Berger et al. 2007).

### Concentration–response relationship carbamazepine

**Figure 4:**
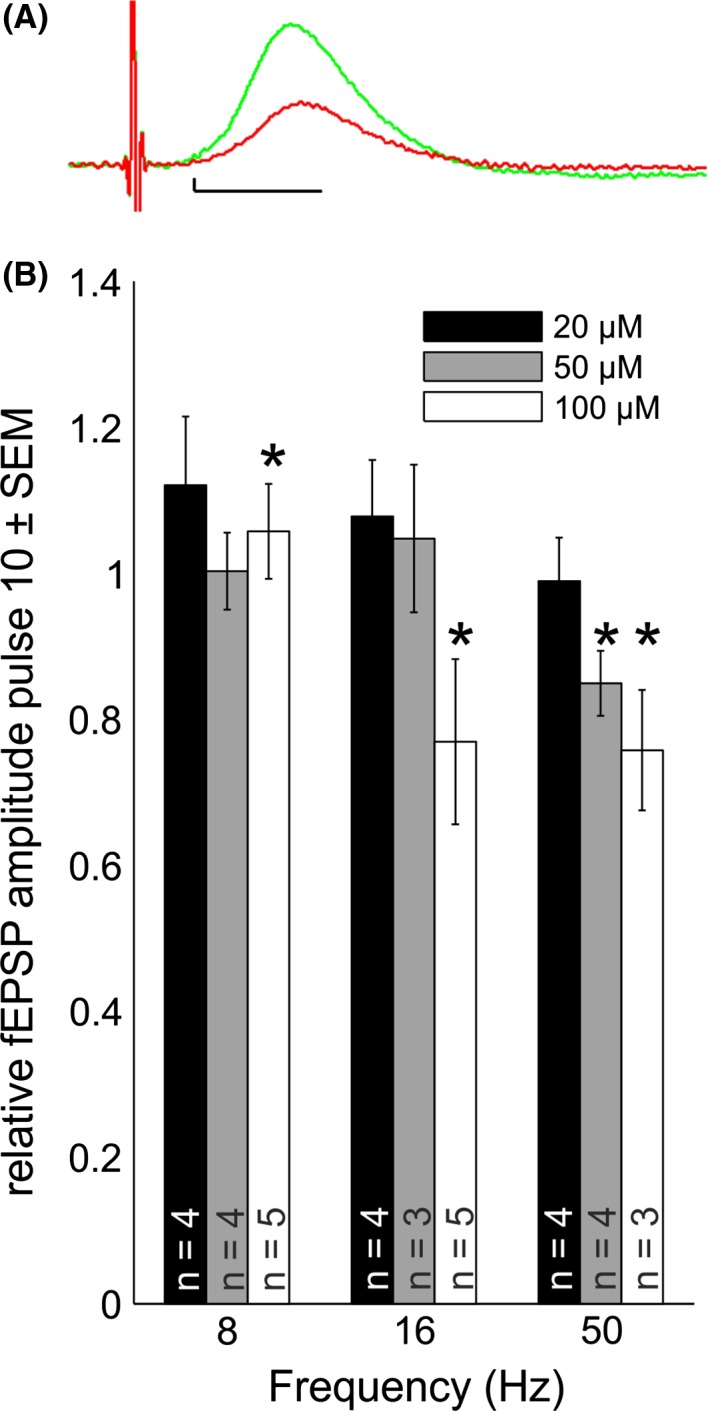
The concentration–response effect of carbamazepine in the rat dentate gyrus. (A) Example of a field potential in response to the 10th pulse of a 16 Hz pulse train recorded in the granule cell layer of the DG. The green trace is control condition, red trace is after 20‐min bath application of 100 *μ*mol/L carbamazepine. Scale: 5 msec, 0.1 mV. (B) The concentration–response effect of carbamazepine (20, 50, and 100 *μ*mol/L) on the relative amplitude of the field potential amplitude (fEPSP) in the granule cell layer of the dentate gyrus tested at the 10th pulse of frequency trains of 8, 16, and 50 Hz. * indicate a significant difference between the control and the CBZ condition.

The concentration–response relation of CBZ was investigated in the rat DG with field potential recordings. Stimulus trains of 10 pulses at 8, 16, and 5 Hz at 100% of the maximum stimulus intensity were applied to the outer molecular layer of the DG. Stimulus‐evoked field potentials (fEPSP) were recorded in granule cell layer of the DG from healthy rats. The fEPSP were obtained in the control situation and after 20 min wash‐in of CBZ at 20, 50, and 100 *μ*mol/L (Fig. 4A). The concentration–response relationship for CBZ was explored by determining the % inhibition of the fEPSP peak amplitude at the 10th pulse after CBZ application (Fig. 4B). Paired *t*‐tests were conducted to compare the fEPSP amplitude between the control situation and CBZ, for all tested frequencies. Bonferroni correction was performed to correct for multiple testing and the *α*‐level was set at 0.017. Significant effects of CBZ were detected for 50 *μ*mol/L CBZ at 16 Hz (*P* = 0.0065 – *t* = −5.6) and for 100 *μ*mol/L CBZ at 8, 16, and 50 Hz (*P* < 0.0005 – *t* = 16.3, *P* = 0.007 – *t* = 6.8 and *P* = 0.01 – *t* = 9.2, respectively).

### Effect of carbamazepine in the rat dentate gyrus

**Figure 5:**
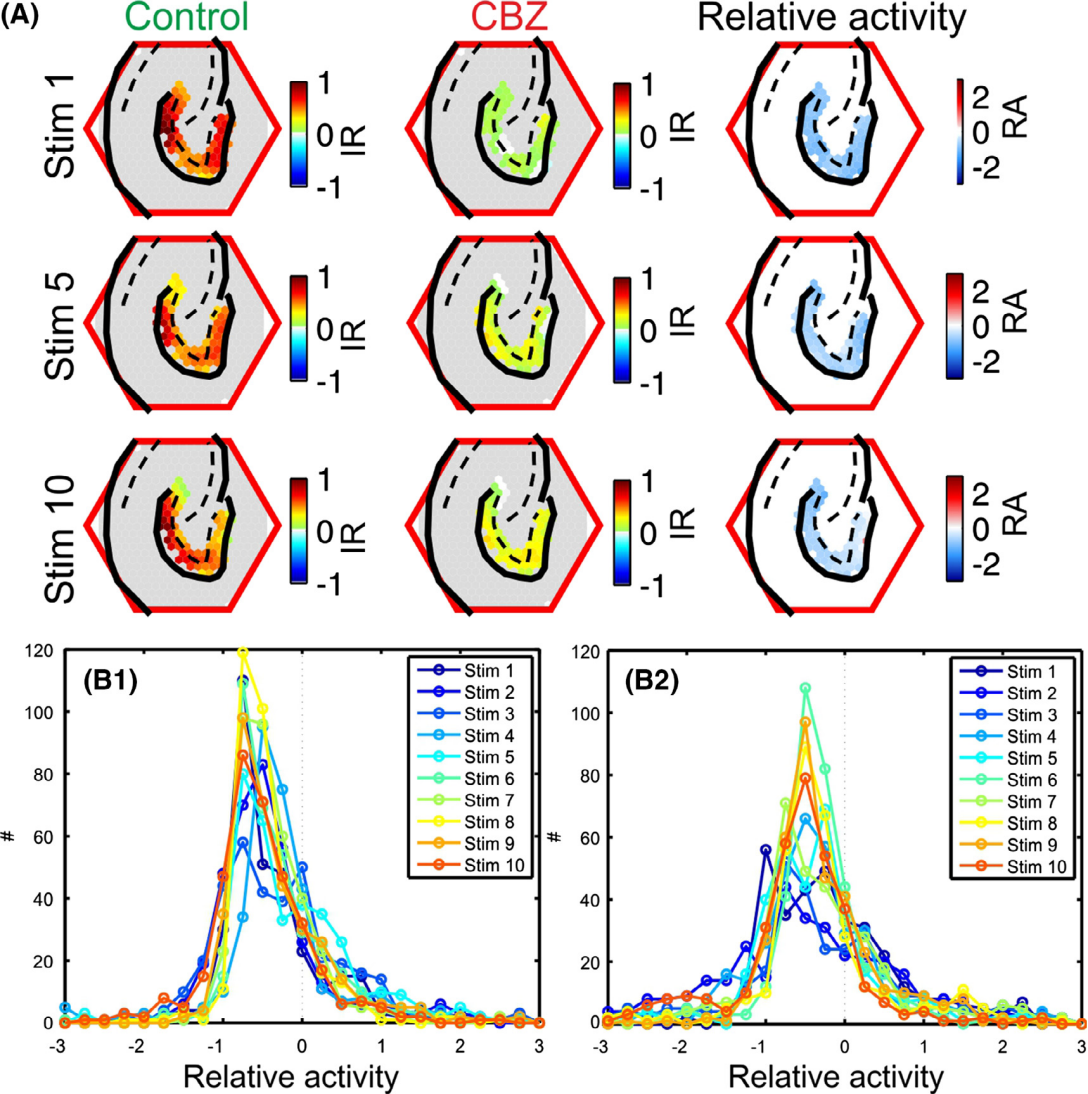
Effect of carbamazepine in rat dentate gyrus. (A) Spatial dynamics of the integrated response recorded in the molecular layer of the DG in response to a stimulus train at 16 Hz (500 *μ*A) in the control condition (first column, “Control”) and 100 *μ*mol/L CBZ (second, “CBZ”). The RA (relative activity) between the CBZ and control response is represented in the third column, RA = (CBZ – Control)/Control. (B) The distribution of the relative activity in the molecular layer of the dentate gyrus for all slices (*n* = 7), for 8 Hz (B1) and 16 Hz (B2) stimulus frequencies.

Stimulus trains of 10 pulses at 8 and 16 Hz were applied to the outer molecular layer of the rat DG and the evoked responses were measured with VSD imaging in the control situation, 20 min after wash‐in of 100 *μ*mol/L CBZ and 20 min after wash‐out of CBZ. A typical example of the spatial dynamics of the integrated VSD response is shown in Figure 5A in response to stimulus pulses 1, 5, and 10 applied at 16 Hz (500 *μ*A). In the control condition (ACSF), all pulses of the stimulus train activated the entire molecular layer of the DG, while application of 100 *μ*mol/L CBZ decreased the integrated responses. Typically, the relative activity integrated response (CBZ – control/control) showed a decrease in the integrated response in the entire molecular layer of the DG in the typical example (Fig. 5A – third column). This relative activity was calculated for all diode channels in the molecular layer of all seven rat slices (Fig. 5B). The distribution of the relative activity is shifted toward negative values, indicating that CBZ reduced the size of the integrated response for 8 and 16 Hz (Fig. 5B). There was a significant effect of CBZ on the relative activity on all 10 pulses for 8 as well as 16 Hz (Two‐way ANOVA with repeated measures performed on the data from all channels in the molecular layer; *P* < 0.01).

**Figure 6:**
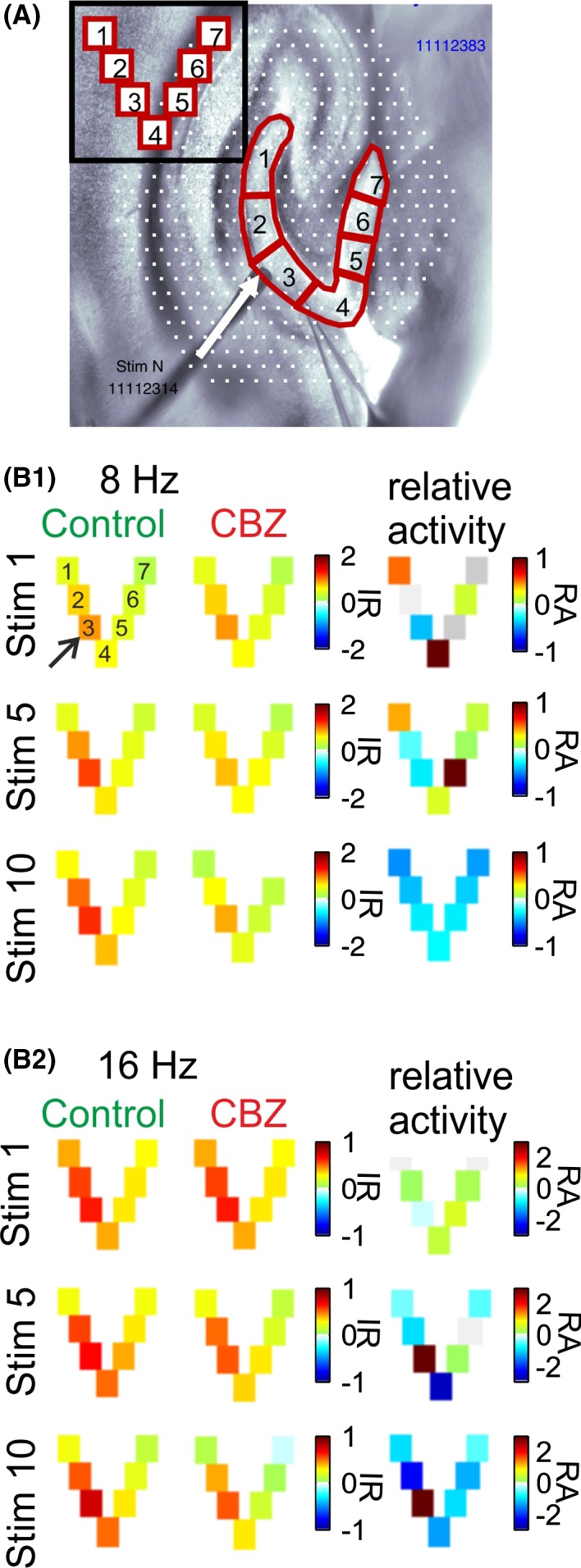
The spatial effect of carbamazepine in the rat dentate gyrus. (A) Image of a rat hippocampal slice. The small white squares represent the location of the 464 photodiode channels; the position of the bipolar stimulation electrode is indicated by the white arrow. The molecular layer of the dentate gyrus is divided into seven fields. The numbers of the fields represent the: 1. Inner blade – tip; 2. Inner blade – middle part; 3. Inner blade – bottom part; 4. Crest; 5. Outer blade – bottom part; 6. Outer blade – middle part; and 7. Outer blade – tip. For the analysis, the channels included within one field were averaged and these values were used for averaging over slices and schematically represented (“V”‐shape – inset). (B) The average of the integrated responses (IR) within each of the seven defined fields (A) was calculated and averaged over seven slices for each evoked response following the 10 pulses applied at 8 Hz (B1) or 16 Hz (B2). The columns represent the control condition (“Control”), 100 *μ*mol/L CBZ application for 20 min (“CBZ”) and the relative activity (RA) between control and CBZ. The averaged integrated response values (column 1–2) are represented in color code at the corresponding field in the DG. The black arrow in the top left panel in B1 shows the location of the stimulation electrode. The numbers represent the fields shown in A.

The similarities in the rat DG shape enabled us to average the integrated responses over slices and investigate the spatial dynamics of CBZ (Fig. 6). The mean value of the integrated response for each field were averaged over all tested slices (Fig. 6B). The highest value of the integrated response was always observed near the stimulation electrode (field 3 of the DG in Fig. 6A). To determine the CBZ effect, the relative activity was calculated. Application of 100 *μ*mol/L CBZ significantly reduced the response amplitude for 8 and 16 Hz (Three‐way ANOVA with repeated measures, *P* < 0.05 for both 8 and 16 Hz) in all fields (*P* < 0.01 for both 8 and 16 Hz). A significant effect of pulse number was only observed for 16 Hz (*P* < 0.01; Fig. 6B), although a significant interaction between DG field and pulse number was observed for both 8 and 16 Hz (*P* < 0.01).

## Discussion

Carbamazepine is a commonly used AED, which blocks epileptic activity mainly by exerting a use‐dependent block on voltage‐activated Na^+^ channels (Rogawski and Loscher 2004; Sun et al. 2006; Qiao et al. 2014). In this study, we used VSDi to investigate whether CBZ is still capable to modulate electrically evoked responses in DG brain slices obtained from pharmacoresistant patients. Electrical stimulation of the DG in the control situation evoked responses in human hippocampal brain slices and the amplitude of the evoked responses increased with increasing stimulus intensity. The strongest largest response was located near the stimulation electrode, probably because the connectivity of the slice was best preserved in this part; and connectivity to other parts of the epileptic hippocampus could have been compromised by sclerosis. The spatial extent of the response seemed independent of the stimulus intensity. This is corroborated by a study in human neocortical slices, which describes that the stimulus intensity had no influence on the lateral spread (Straub et al. 2003). Similar results were found in our rat DG slices. Even low‐intensity stimulation of the perforant path activated the complete molecular layer of the DG and increasing the stimulus intensity enhanced the amplitude. Moreover, Ang et al. (2006) showed that the spatial extent of the stimulus‐evoked activation of the DG did not differ between control and epileptic rats (Ang et al. 2006).

The application of 100 *μ*mol/L CBZ reduced the amplitude of the VSD responses in the molecular layer of the DG in rats as well as in human slices. The amplitude of the responses did not fully recover to the control level after wash‐out of CBZ; most likely complete wash‐out of the high concentration of 100 *μ*mol/L CBZ would take a very long time due to the lipophilic nature of the drug (Hood et al. 1983; Cunha et al. 2002) although a decline of the slice quality over time cannot be excluded.

The concentration–response data showed that 100 *μ*mol/L CBZ is a concentration that affects the fEPSP amplitude in the rat DG and we used this concentration of CBZ for the VSD experiment. The CBZ concentration tested in our study could be considered as high, as therapeutic concentrations of CBZ in serum and cerebrospinal fluid levels in patients ranged between 12 and 50 *μ*mol/L (Johannessen et al. 1976; Friis et al. 1978; Strandjord et al. 1980; Semah et al. 1994; Neels et al. 2004; Rambeck et al. 2006). However, EC50 values of CBZ reported in the literature range between 131 and 263 *μ*mol/L (Schumacher et al. 1998; Cunha et al. 2002; Teriakidis et al. 2006; Giustizieri et al. 2008) which are higher compared to the concentration of CBZ used in our study. Moreover, our observation that application of 100 *μ*mol/L CBZ reduced the amplitude of the evoked response in slices from pharmacoresistant patients is confirmed by the results of Jandova et al. (2006). They reported that 100 *μ*mol/L CBZ reduced the response amplitude or even terminated the experimentally induced epileptic activity in cells of the DG in some slices of the MTLE group, while 50 *μ*mol/L only reduced the signal amplitude in DG slices of the tumor group and not in the slices of the MTLE group (Jandova et al. 2006) or had no effect at all in hippocampal and cortical slices (Sandow et al. 2015). Reckziegel and colleagues reported a reduced maximal Na^+^ current amplitude with increasing CBZ concentration (15–320 *μ*mol/L) and a saturated shift in the voltage‐dependent steady‐state inactivation in the hyperpolarizing direction in human DG cells acutely isolated from the focal epileptic area for CBZ concentrations of 100 *μ*mol/L and higher (Reckziegel et al. 1999).

Carbamazepine binds to the *α*‐subunit of voltage‐gated Na^+^‐channels in a use‐dependent manner (Rogawski and Loscher 2004; Qiao et al. 2014). In our results, the evoked activity was reduced in response to all 10 pulses of the stimulus train, in human as well as in rat slice preparations. This could be explained by the relatively depolarized resting membrane potential of the DG granule cells of humans (Williamson et al. 1995; Isokawa et al. 1997; Stegen et al. 2012) and rats (Staley et al. 1992; Isokawa et al. 1997; Kobayashi et al. 2004). This implies that, at rest, a considerable fraction of the Na^+^ channels is in the inactivated state (Qiao et al. 2014) and therefore CBZ can already bind to these inactivated channels before the first stimulus of the train is applied. However, it cannot be excluded that CBZ also binds to other channels, like presynaptic Ca^2+^ channels (Schumacher et al. 1998; Sitges et al. 2007; Giustizieri et al. 2008), L‐type Ca^2+^ channels (Willow et al. 1985; Ambrosio et al. 1999), NMDA, or kainate receptors (Cai and McCaslin 1992; Cunha et al. 2002). CBZ binding to these targets most likely lacks the use‐dependent characteristic. A third explanation could be that the duration of the interstimulus interval between subsequent pulses affects the recovery from fast inactivation (Reckziegel et al. 1999). Interstimulus intervals of 50 msec and longer resulted in Na^+^ channel availability close to 100% (Ellerkmann et al. 2001). However, Remy et al. (2003) showed significantly blocked Na^+^ currents for 100 msec intervals. The stimulus frequencies (8 and 16 Hz) used in our experiments resulted in interstimulus intervals of 125 and 62.5 msec, suggesting that the frequencies used in this study are probably less discriminative to show the use‐dependent effect.

## Conclusion

All stimulus evoked responses in human and rat DG were sensitive to 100 *μ*mol/L CBZ, although the patients who underwent surgery in this study were all clinically diagnosed as CBZ pharmacoresistant. Apparently the CBZ pharmacoresistance does not imply a complete lack of CBZ sensitivity; it is mainly based on a lack of sufficient seizure control. CBZ also modulated the spatial dynamics of stimulus evoked activity in a similar manner in patient material as in slices obtained from control rats. These results support the view that in situ drug administration in patients is a promising way in epilepsy treatment.

## Conflict of Interest

None declared.
